# First draft genome sequence of *Candidozyma auris* from a human clinical isolate in a major metropolitan hospital in Lima, Peru

**DOI:** 10.1128/mra.00538-25

**Published:** 2026-02-04

**Authors:** Diego Cuicapuza, Fernando Soto-Febres, Edgar Neyra, Beatriz Bustamante

**Affiliations:** 1 Laboratorio de Genómica Microbiana, Facultad de Ciencias e Ingeniería, Universidad Peruana Cayetano Heredia 33216, Lima, Peru; 2 Facultad de Medicina, Universidad Peruana Cayetano Heredia 33216, Lima, Peru; 3 Servicio de Infectología, Hospital Nacional Guillermo Almenara Irigoyen – EsSalud, Lima, Peru; 4 Unidad de Genómica, Facultad de Ciencias e Ingeniería, Universidad Peruana Cayetano Heredia 33216, Lima, Peru; 5 Laboratorio de Micología Clínica, Instituto de Medicina Tropical "Alexander von Humboldt," Universidad Peruana Cayetano Heredia 33216, Lima, Peru; University of California Riverside, Riverside, California, USA

**Keywords:** *Candidozyma auris*, emerging pathogens, antifungal susceptibility testing, whole-genome sequencing, bioinformatics

## Abstract

We present the draft genome sequence of a fluconazole-resistant *Candidozyma auris* isolate obtained from a Peruvian referral hospital. *C. auris* is an emerging pathogenic yeast of significant public health concern due to its antifungal resistance, particularly in patients with comorbidities.

## ANNOUNCEMENT

*Candidozyma auris* is an emerging, multidrug-resistant fungal pathogen that has raised significant concern due to its rapid global spread and the high mortality rates associated with healthcare-related outbreaks (1, 2). Although first identified in 2009, *C. auris* has been reported in more than 30 countries, highlighting the need for further studies to understand its genomic epidemiology and resistance mechanisms, particularly in intensive care units (3–5). In Peru, the first *C. auris* outbreak was reported in August 2020 during the first wave of the COVID-19 pandemic at the Hospital Nacional Guillermo Almenara Irigoyen, a referral health center located in Lima (6).

*C. auris* isolate encoded as 8H was obtained from a post-surgical left femoral chronic osteomyelitis sample from a 32-year-old patient. An intramedullary tissue culture was performed in standard culture media; after incubation and positive growth, it was identified as *C. auris* with the YST ID card of the Vitek 2 Compact instrument (bioMérieux, France). The study was evaluated and approved by the Institutional Research Ethics Committee (CIEI) of Universidad Peruana Cayetano Heredia (SIDISI No. 217908). The genomic DNA of the isolate was extracted using the column-based Quick-DNA Fungal/Bacterial Miniprep Kit (Zymo Research) following the manufacturer’s instructions. Sequencing libraries were prepared using the Illumina DNA Prep kit (Illumina, Inc., San Diego, CA, USA) and sequenced on an Illumina NovaSeq X Plus instrument, generating 29,464,068 paired-end 150-bp reads and a mean of 340× genome coverage.

Raw reads were assessed with FastQC v0.12 (7), processed with Fastp v0.23.4 using default parameters (8), and *de novo* assembled with SPAdes v.3.15.5 (9), with the option “isolate.” The final assembly was annotated using the Funannotate v.1.8.17 (10) pipeline with default parameters, using the *ab initio* gene prediction tool Augustus v3.5.0 (11). The *C. auris* clade based on Mash distances from reference genomes was predicted with the AuriClass v.0.5.4 program (https://github.com/RIVM-bioinformatics/auriclass). Quality was assessed with Quast v.5.3.0 (12), and the completeness of the assembled genome was evaluated using Benchmarking Universal Single-Copy Orthologue (BUSCO) v5.8.2 (13) against the databases “fungi_odb10.” Mutations associated with antifungal resistance were identified using the MARDy (14) and AFRbase (15) databases.

**Fig 1:**
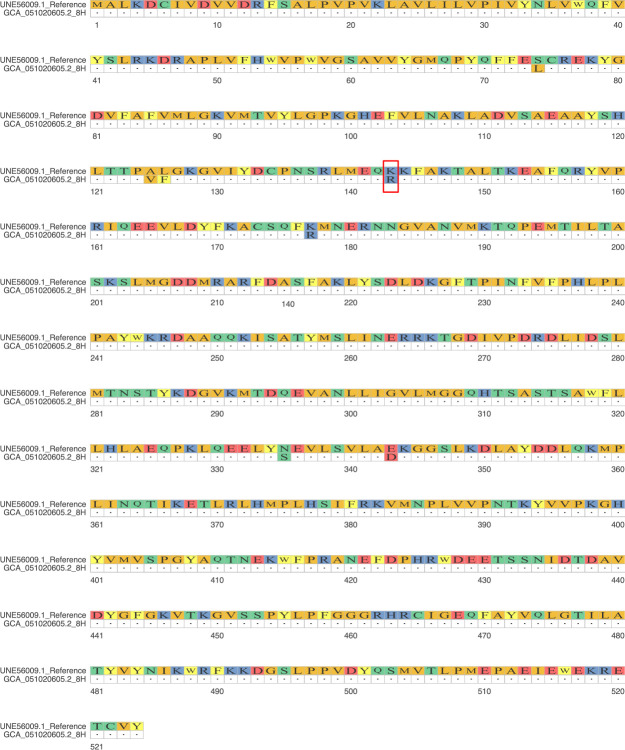
Amino acid substitutions in isolate 8H compared with the reference sequence ERG11 (UNE56009). The red rectangle highlights the amino acid substitution observed in isolate 8H, specifically the K143R mutation. Sequence alignment and visualization were performed using ggmsa v1.14.1 (http://yulab-smu.top/ggmsa/) in R v4.5.0.

The antifungal susceptibility test showed susceptibility to voriconazole (MIC: 0.5 µg/mL), micafungin (MIC ≤ 0.06 µg/mL), caspofungin (MIC ≤ 0.12 µg/mL), and amphotericin B (MIC: 1 µg/mL), but resistance to fluconazole (MIC: 16 µg/mL) according to Vitek 2–specific wild-type upper limit values. This fluconazole resistance was consistent with the K143R point mutation identified in the ERG11 gene, confirming the concordance between phenotypic and genotypic findings (Fig. 1). The isolate was classified as clade IV. The assembled genome size was 12,319,269 (12.3 MB), with 321 contigs larger than 1000 bp (N50 138,402), a GC content of 45.1%, and BUSCO completeness of 95.8% (95.6% single, 0.2% duplicated).

This study presents a whole-genome sequencing and analysis of a fluconazole-resistant *C. auris* isolate from the South American clade (Clade IV), obtained from a patient in Peru. This analysis will enhance the global understanding of this pathogen’s epidemiology and support the development of targeted treatment strategies.

## Data Availability

The draft genome assembly and the raw reads of *Candidozyma auris* 8H have been submitted to NCBI under BioProject PRJNA1264495, with the assembly available in GenBank under accession number JBNYWV000000000.2. The corresponding annotated files (GBK and GFF3) are also deposited on Zenodo under https://doi.org/10.5281/zenodo.17459964.
